# Impact of Gastric pH on Milk Protein Hydrolysis: A Pilot In Vitro Study Using Pediatric Human Gastric Juice in the Context of Infant Digestive Physiology

**DOI:** 10.3390/children13050595

**Published:** 2026-04-24

**Authors:** Maria Del Nogal Avila, Marta Soria López, Isabel Sánchez-Vera, Rosa Plaza-Clavero, Daniel Cabello-Rivera, Karen Knipping, Alejandro López-Escobar

**Affiliations:** 1 Instituto de Medicina Molecular Aplicada (IMMA) Nemesio Díez, Facultad de Medicina, Universidad San Pablo-CEU, CEU Universities, Urbanización Montepríncipe, 28660 Boadilla del Monte, Spain; maria.nogalavila@ceu.es (M.D.N.A.); isanver@ceu.es (I.S.-V.); rosaplazaclavero@gmail.com (R.P.-C.); daniel.cabellorivera@ceu.es (D.C.-R.); 2 Pediatrics Department, HM Hospitales, 28938 Madrid, Spain; soria.marta@gmail.com; 3 Ausnutria B.V., 8025 Zwolle, The Netherlands; karen.knipping@ausnutria.nl; 4 Facultad de Ciencias de la Salud, Universidad Internacional de la Rioja (UNIR), 28224 Pozuelo de Alarcón, Spain

**Keywords:** goat milk formula, infant digestion, gastric pH, gastroesophageal reflux (GERD), human gastric juice, milk protein hydrolysis, α-lactalbumin, pediatric nutrition

## Abstract

**Highlights:**

**What are the main findings?**

- Goat milk infant formula caseins and α-lactalbumin exhibit greater apparent hydrolysis rates under these experimental conditions than cow’s milk infant formula when using pediatric human gastric juice at elevated pH levels.
- Human milk proteins, particularly lactoferrin and albumin, demonstrate high structural stability compared to infant formulas across a physiological gastric pH gradient (2.5 to 6.0).

**What are the implications of the main findings?**

- The higher observed hydrolysis of goat milk infant formula under low-acid conditions suggests further investigation in clinical settings involving infants with gastroesophageal reflux disease undergoing acid-suppressive therapy.
- The observed resistance to digestion in human milk proteins supports their evolutionary role in preserving bioactivity and functional integrity during the early stages of infant gastric transit.

**Abstract:**

Background/Objectives: Gastroesophageal reflux disease (GERD) is prevalent in infants and frequently managed with acid-suppressive medications that elevate gastric pH. This pilot study aimed to evaluate how varying gastric pH levels (2.5, 4.0 and 6.0) influence the hydrolysis of milk proteins in human milk (HM), cow’s milk-based infant formula (CMF), and goat milk-based infant formula (GMF). Methods: Samples were subjected to a 30 min in vitro gastric digestion using pediatric human gastric juice obtained from clinical donors. Protein degradation was analyzed via SDS-PAGE densitometry, comparing digested aliquots to undigested controls. Results: At pH 2.5, caseins were highly digested in all samples, especially in HM and GMF. At pH 4.0, GMF displayed an apparent 51% greater casein degradation relative to CMF and HM in this pilot analysis. α-lactalbumin degradation was markedly higher in GMF at all pH levels; notably, at pH 4.0 and 6.0, only GMF exhibited digestion of this protein. Albumin showed almost complete degradation in HM and GMF at pH 2.5, and GMF maintained greater degradation at higher pH levels. β-lactoglobulin (absent in HM) was better digested in GMF at pH 2.5, whereas CMF showed higher hydrolysis observed at pH 4.0 and 6.0. Lactoferrin digestion was most efficient in HM and GMF at pH 2.5, with no differences observed at higher pH levels. Conclusions: These preliminary findings suggest that GMF may offer digestive advantages for infants with GERD under pharmacological acid suppression, particularly regarding casein and α-lactalbumin breakdown at higher pH. The distinct digestion kinetics of CMF and GMF at different pH levels provide a physiological basis for targeted infant feeding strategies. Further large-scale studies are required to validate these exploratory observations.

## 1. Introduction

Breastfeeding remains the gold standard for infant nutrition; however, safe and effective alternatives are necessary when supplementation or replacement becomes essential. Most commercially available infant formulas are based on cow’s milk (CM) or soy protein. Nonetheless, goat milk-based infant formulas (GMF) have gained popularity due to their comparable support for infant growth and endorsement by the European Food Safety Authority (EFSA) and the Spanish Association of Pediatrics [1,2,3,4].

Several studies suggest that goat milk (GM) exhibits different digestion kinetics than CM [5,6,7], likely due to compositional differences in caseins. Notably, the casein profile of GM more closely resembles that of human milk (HM), potentially altering the rate of protein degradation [8,9,10]. Furthermore, research using human gastric and duodenal juices has shown that GM-derived β-lactoglobulin is more digestible than its CM counterpart [5,10]. GM consumption has also been associated with stool and microbiome characteristics similar to those observed in breastfed infants [11,12].

GM proteins are structurally more similar to those found in HM [8,10]. Additionally, GM produces curds with lower tension (36 g) than CM (70 g) [9,13,14]. Lower curd tension indicates a more fragile coagulate, associated with improved digestibility and accelerated gastric emptying. In vitro trypsin-based assays have supported these distinct protein hydrolysis patterns [15]. Notably, many studies rely on commercial porcine enzymes, which may overestimate proteolysis [16]. Eriksen et al. [16] emphasized that human gastric juices (HGJ) reflect in vivo neonatal digestion more accurately. This is particularly relevant when considering the gastric resistance of certain bioactive proteins [17,18] and the specific acid secretion patterns in infants [19,20].

Gastroesophageal reflux (GER) is common in infancy, but when complications arise, it is diagnosed as gastroesophageal reflux disease (GERD) [21,22,23], affecting approximately 8% of infants [24]. Although it often resolves spontaneously, persistence is reported in many cases [25,26,27]. Anatomical immaturity and ineffective peristalsis contribute to the high incidence in neonates [28,29]. Diagnosis relies on clinical history and complementary tests to distinguish physiological GER from GERD [23,30,31].

The primary goal in managing GERD is to alleviate symptoms and prevent mucosal injury, often using pharmacological treatments that target gastric pH [32]. However, these medications significantly alter gastric acidity, which may impact protein hydrolysis and overall nutrient absorption, as seen in cases of nutrient-related complications like hypophosphatemic rickets [33]. Recent studies have further explored these dynamics under simulated infant conditions [34,35], highlighting the need to understand how bioactive proteins like lactoferrin [36,37] resist digestion across different matrices [38].

We hypothesized that GMF, due to its unique protein structure and curd-forming properties, would exhibit a higher susceptibility to pepsin-mediated hydrolysis compared to CMF under the elevated gastric pH conditions typical of infants receiving acid-suppressive therapy.

Despite advances in formula composition, there is a critical lack of evidence regarding how milk protein matrices interact with the specific proteolytic environment of the infant stomach when acidity is pharmacologically compromised. Current models predominantly rely on porcine enzymes, which do not accurately replicate the unique kinetics of human gastric juice (HGJ) in a pH-dependent context. Our results provide preliminary evidence that GMF exhibits a more efficient protein degradation profile at higher pH levels compared to CMF, particularly for caseins and α-lactalbumin. These findings suggest that formula selection could be a key nutritional strategy for infants undergoing acid-suppressive therapy.

## 2. Materials and Methods

### 2.1. Milk Samples and Preparation

An in vitro study was performed to evaluate the effect of pH on protein digestion in three types of milk: HM, GMF (Kabrita^®^ stage 1, Ausnutria, Zwolle, The Netherlands), and CMF (Blemil Plus Forte^®^ Stage 1, Ordesa, Barcelona, Spain). Both formulas are fortified with whey to reach a whey/casein ratio of 60:40. GMF and CMF were reconstituted according to the manufacturers’ instructions (Supplementary Table S1). To simulate various clinical scenarios, such as infants under acid-suppressive therapy, protein digestion was analyzed at three pH levels: 2.5 (optimal pepsin activity), 4.0 (representing the typical postprandial infant stomach), and 6.0 (simulating the effect of pharmacological acid suppression).

### 2.2. Collection of Biological Samples

#### 2.2.1. Human Milk Collection

HM was obtained from a healthy donor mother (4 months postpartum) who provided informed consent. The sample was mid-feed milk, expressed between 08:00 and 10:00 a.m. to minimize diurnal protein fluctuations. The donor followed a standard Mediterranean diet with no medication use. Samples were stored at −20 °C and underwent only one freeze–thaw cycle before analysis. Samples were transported on dry ice to the Department of Basic Medical Sciences (Facultad de Medicina, Universidad CEU San Pablo) and stored in aliquots at −20 °C.

#### 2.2.2. Human Gastric Juice Collection

Due to the significant ethical and logistical challenges in obtaining gastric juice from healthy pediatric donors, this study was designed as a pilot investigation using a single, well-characterized donor to ensure a ‘pure’ physiological matrix for this exploratory analysis. HGJ was obtained from a single pediatric donor, a 14-month-old male undergoing elective endoscopy. The donor was selected based on the absence of gastrointestinal conditions or medications (e.g., proton pump inhibitors) that could alter gastric secretion profiles. Informed consent was obtained from the legal representative. The sample was collected at Hospital Universitario HM Puerta del Sur, centrifuged (4500× *g*, 10 min, 4 °C) to remove mucus and debris, and stored at −20 °C until use.

### 2.3. Pepsin Activity Measurement

The proteolytic capacity of the HGJ was standardized via hemoglobin degradation assays. Samples were incubated with 2.5% hemoglobin (Sigma-Aldrich, St. Louis, MO, USA) for 10 min at 37 °C. The reaction was stopped with 5% trichloroacetic acid, filtered (0.45 µm), and absorbance was measured at 280 nm (Biophotometer Plus, Eppendorf, Germany). Porcine pepsin (Sigma-Aldrich) was used as a positive control. Activity was expressed in Units (0.4 U/50 µL), adding the same amount of solution to identical volumes of each of the milks, ensuring a uniform and consistent enzyme-to-substrate ratio across all digestion trials.

### 2.4. In Vitro Gastric Digestion Protocol

Digestion was performed by adding the standardized HGJ to the milk samples. The pH was adjusted to 2.5, 4.0, or 6.0 using 1N HCl. The mixture was incubated for 30 min at 37 °C, reflecting the initial phase of infant gastric transit. The reaction was terminated by increasing the pH to 8.0 with 1N NaOH. Undigested milk samples (pre-enzymatic addition) served as baseline controls.

This study specifically focused on the gastric phase of digestion, as this is the primary site of action for acid-suppressive medications and the anatomical origin of GERD-related complications.

### 2.5. SDS-PAGE Electrophoresis and Densitometry

To analyze the protein degradation profile, equal volumes of each sample were loaded to maintain a constant matrix ratio. Samples were mixed with 4x sample buffer (160 mM Tris-HCl pH 6.8, 8% SDS, 65 mM DTT, 30% glycerol) and denatured at 95 °C for 5 min. Aliquots (15 µL of a 1/8 dilution) were loaded into 15% polyacrylamide gels. Electrophoresis was run at 200 V for 50 min (Mini-PROTEAN Tetra Cell, Bio-Rad, Hercules, CA, USA). Gels were stained with GelCode Blue (Thermo Scientific, Waltham, MA, USA) and scanned (ChemiDoc, Bio-Rad). Densitometric analysis of protein bands (caseins, α-lactalbumin, β-lactoglobulin, albumin, and lactoferrin) was performed using Image Lab software (Bio-Rad, 6.1.0 build 7), with results expressed as a percentage of the respective undigested control.

Numerical values are expressed as the percentage of protein hydrolysis (loss of intact protein) relative to undigested control after 30 min of incubation. This parameter, calculated by comparing the band density of the digested sample against its respective undigested control, serves as a primary proxy for the extent of protein hydrolysis in each milk matrix. It should be noted that SDS-PAGE densitometry is a semi-quantitative method that monitors the disappearance of intact protein bands; it does not account for the release of low-molecular-weight peptides.

### 2.6. Ethical Approval

The study was conducted in accordance with the Declaration of Helsinki and approved by the HM Hospitales Drug Research Ethics Committee (Protocol Code: 20.02.1522-GHM). Written informed consent was obtained from the participants’ legal guardians.

## 3. Results

**Figure 1:**
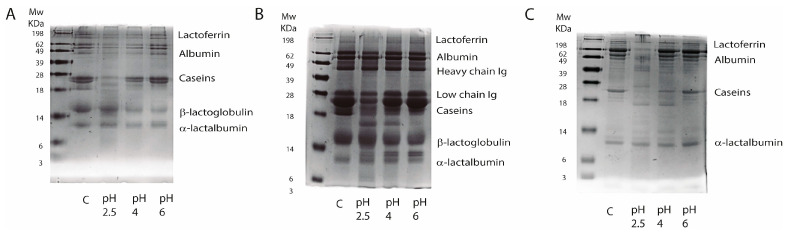
Polyacrylamide gel electrophoresis after 30 min of digestion with gastric juice from donor of cow’s milk-based formula protein (**A**), goat milk-based formula proteins (**B**) and human milk proteins (**C**) under different pH conditions. C is an undigested control sample.

The protein degradation patterns of HM, CMF and GMF were identified by molecular weight using SDS-PAGE and compared across a pH gradient (2.5, 4.0, and 6.0). The densitometric analysis of the protein bands, expressed as a percentage of residual intact protein (band density) relative to the undigested control, revealed distinct pH-dependent hydrolysis kinetics for each matrix (Figure 1A–C).

### 3.1. Casein Digestion Patterns

**Figure 2:**
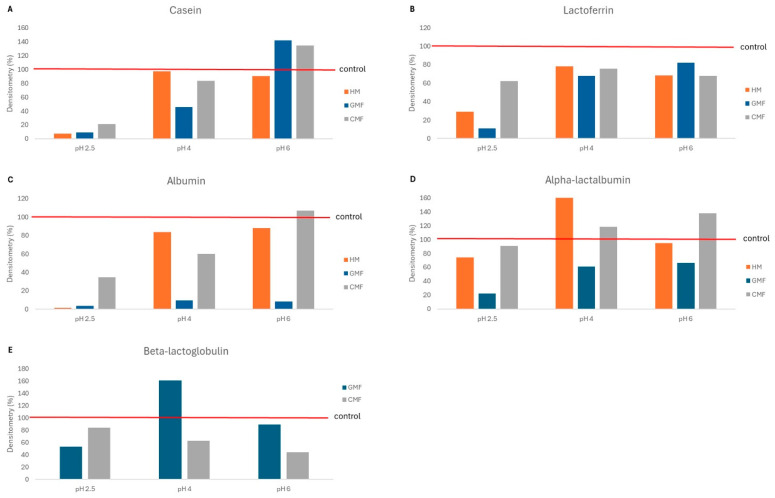
Percentage of band density changes relative to undigested samples under different pH conditions. The undigested sample is indicated as a 100% reference line in red. (**A)**, casein; (**B**) Lactoferrin; (**C**) Albumin; (**D**) α-lactalbumin; (**E**) β-Lactoglobulin.

Caseins bands appeared highly sensitive to acidic conditions, showing extensive hydrolysis at pH 2.5 in all milk types, with residual band densities dropping to 21%. At this optimal pH, the reduction in band density appeared most pronounced in HM and GMF compared to CMF. Crucially, at pH 4.0 (simulating moderate acid suppression), GMF was observed to have an apparent 51% higher casein degradation than CMF and HM. As pH increased to 6.0, CMF caseins showed an apparent increase in density (134.5%), while GMF caseins exhibited the widest range of variation (from 9.2% at pH 2.5 to 141.8% at pH 6.0). In contrast, HM caseins maintained relatively higher stability across the pH range (Figure 2A, Table 1).

### 3.2. Whey Protein Hydrolysis: α-Lactalbumin and β-Lactoglobulin

α-Lactalbumin showed marked interspecies differences in digestibility. At pH 2.5, degradation was 51% greater in GMF than in HM. Notably, at higher pH values (4.0 and 6.0), only GMF exhibited greater apparent α-lactalbumin reduction (residual density below 66%), whereas no digestion was observed in CMF or HM, highlighting the unique pH sensitivity of goat milk whey proteins (Figure 2D, Table 1).

Regarding β-lactoglobulin (absent in HM), GMF showed lower stability at pH 2.5 (52.9% retention) compared to CMF (84.2% retention). Notably, at pH 4.0, GMF β-lactoglobulin showed an apparent density of 161.2% relative to the control. This result reflects a common methodological limitation of SDS-PAGE densitometry, where digestion fragments from larger proteins (e.g., caseins) co-migrate and stack at the same molecular weight as β-lactoglobulin, artificially inflating the signal (Figure 2E, Table 1).

### 3.3. Bioactive Proteins: Lactoferrin and Albumin

Lactoferrin hydrolysis followed a pattern similar to caseins at pH 2.5, being more efficient in HM and GMF than in CMF (Figure 2B). At this acidic pH, GMF lactoferrin was highly prone to hydrolysis (10.8% retention), while HM lactoferrin remained the most stable (78.3% at pH 4.0). At higher pH levels, no differences were found among the three milk types (Figure 2B and Table 1).

Albumin digestion at pH 2.5 was nearly complete in HM and GMF (<4% residual density). Under low-acid conditions (pH 4.0 and 6.0), albumin degradation remained more pronounced in GMF (<10% density), whereas CMF and HM retained considerably higher amounts of intact albumin, indicating lower proteolytic susceptibility in these matrices (Figure 2C and Table 1).

## 4. Discussion

This pilot study is intended as a hypothesis-generating exercise, identifying specific trends in protein hydrolysis that require validation in larger, multi-donor cohorts. We have investigated the in vitro gastric digestion of major milk proteins from CMF, GMF, and HM under varying pH conditions, highlighting interspecies variations in hydrolysis kinetics. Our findings demonstrate that gastric pH is a critical determinant of protein degradation, with GMF exhibiting higher hydrolysis observed under mildly acidic conditions (pH 4.0) compared to CMF.

The selected pH values reflect key clinical and physiological states: pH 4.0 simulates the typical postprandial gastric environment in neonates and infants [23,29,37]; pH 6.0 reflects the elevated pH observed under pharmacological acid suppression (e.g., PPI therapy) [30,32]; and pH 2.5 serves as a positive control for optimal pepsin activity.

Previous research using porcine enzymes suggested that GMF and CMF have similar breakdown properties [34,35]. However, our study utilizing pediatric HGJ reveals a more nuanced reality. As Eriksen et al. [16] demonstrated, human digestive juices degrade whey proteins less efficiently than porcine enzymes. By using HGJ, our experimental setup more accurately reflects in vivo pediatric digestion, where GMF demonstrated up to 51% higher casein degradation at pH 4.0 compared to CMF. While not directly measured in this study, the observed proteolysis patterns may be influenced by species-specific protein profiles and curd structures reported in the literature. Specifically, the lower αs1-casein and higher β-casein content of GM is known to promote the formation of softer, more hydrated curds, potentially facilitating pepsin access even when acidity is compromised [5,7,9,13,14]. These findings serve to generate hypotheses regarding gastric emptying and nutrient absorption that require further clinical validation.

Regarding bioactive proteins, HM proteins exhibited consistent evolutionary optimization [14,20,38]. Lactoferrin, a key antimicrobial glycoprotein [36], showed high resistance to digestion in HM at pH 4.0 and 6.0, potentially preserving its bioactivity. In contrast, while GMF lactoferrin was highly susceptible to hydrolysis at pH 2.5, GMF showed a distinct advantage over CMF in the degradation of α-lactalbumin at higher pH levels. The unique physicochemical properties of GM proteins—higher solubility and less compact curd formation [6,8]—facilitate enzymatic access, which is clinically relevant for infants with GERD treated with antacids or PPIs [32].

The unconventional 161.2% increase in β-lactoglobulin density observed in GMF at pH 4.0 deserves technical scrutiny. Furthermore, the interpretation of band density must account for the physicochemical behavior of proteins at different pH levels. For instance, the apparent stability of certain bands might be influenced by protein-protein aggregation or the co-migration of large hydrolysis fragments that remain within the same molecular weight range during electrophoresis. While β-lactoglobulin is a known allergen when incompletely digested [17], the distinct degradation kinetics observed in GMF suggest a different antigenic potential that warrants further investigation via mass spectrometry.

### Clinical Implications and Limitations

While these in vitro observations using HGJ provide higher physiological relevance than standard models, they represent a simplified version of the infant gastrointestinal tract. Therefore, these results should be viewed as a biochemical baseline rather than a direct predictor of clinical outcome. The developmental variability of gastric pH in pediatric populations—ranging from pH ≥ 4.0 in preterm neonates to adult levels by age 3 [19,23,29,37]—underscores the importance of these results. While literature suggests that GMF may have benefits in gastric transit [15], our preliminary in vitro findings specifically demonstrate a higher susceptibility of GMF proteins to hydrolysis at pH 4.0, which may generate hypotheses for further clinical research into its role in cases of impaired acid secretion or pharmacological suppression [32,33].

Despite these promising results, this study has limitations inherent to its nature as a communication. First, as a pilot study, we utilized a single pediatric HGJ donor and a single HM sample. While this ensures the use of a “pure” physiological matrix, future studies should use pooled samples to account for inter-individual variability. Second, the in vitro model does not replicate the full complexity of gastric emptying or the intestinal phase. While the use of HGJ improves physiological relevance, it is important to note that this is an in vitro, short-duration study lacking an intestinal phase, absorption data, and clinical outcomes such as infant growth or symptom relief. Third, the reliance on SDS-PAGE provides a semi-quantitative overview; further studies should incorporate OPA methods, LC-MS/MS (Liquid Chromatography-Mass Spectrometry) or proteomics to map specific peptide release. Finally, the authors acknowledge the potential for bias due to industry affiliations. To ensure scientific integrity, all experimental protocols, including pepsin activity standardization and densitometric measurements, were conducted under rigorous academic supervision. The results reported herein reflect the raw analytical observations, independent of the funding source’s commercial interests.

In summary, our results highlight species-specific digestion patterns where GMF exhibits enhanced protein breakdown under low-acid conditions. The observed differences in protein hydrolysis kinetics suggest that GMF could potentially behave differently in the gastric environment of infants with compromised acidity, a hypothesis that warrants further investigation through clinical trials.

## 5. Conclusions

This pilot study provides preliminary evidence that gastric pH is a decisive factor in the hydrolysis kinetics of milk proteins, revealing critical interspecies differences between HM, CMF and GMF. Our results underscore the importance of considering protein digestibility in infants with altered gastric physiology or those undergoing pharmacological acid suppression. Notably, under the conditions of this pilot investigation, GMF caseins and α-lactalbumin appeared more susceptible to degradation under mildly acidic conditions (pH 4.0) compared to CMF when using HGJ. These findings generate the hypothesis that GMF may behave differently regarding gastrointestinal tolerance and nutrient absorption in vulnerable pediatric populations, such as those with GERD. However, due to the single-donor design and the in vitro nature of the model, these results cannot be used to establish clinical guidelines. Further research with larger cohorts and in vivo studies is necessary to validate these exploratory observations.

## Figures and Tables

**Table 1:** Percentage of protein digestion (loss of intact protein: 100—band density) relative to undigested control samples in different infant formulas and human milk under varying gastric pH conditions. (n = 1). C is an undigested control sample; n.a.: not applicable.

Protein	Cow’s Milk Formula				Goat’s Milk Formula				Human Milk			
	C	pH 2.5	pH 4	pH 6	C	pH 2.5	pH 4	pH 6	C	pH 2.5	pH 4	pH 6
	%	%	%	%	%	%	%	%	%	%	%	%
**Caseins**	0.0	79.0	16. 5	0.0	0.0	90.8	54.2	0.0	0.0	92.9	2.7	9.8
**Lactoferrin**	0.0	37.7	24.4	32.1	0.0	89.2	31.9	17.9	0.0	70.8	21.7	31.8
**Albumin**	0.0	65.6	40.0	0.0	0.0	96.2	90.3	91.7	0.0	98.5	16.3	12.0
**α-lactalbumin**	0.0	8.9	0.0	0.0	0.0	77.6	38.8	33.6	0.0	26.0	0.0	5.0
**β-lactoglobulin**	0.0	15.8	37.0	55.5	0.0	47.0	0	10.8	n.a.	n.a.	n.a.	n.a.

## Data Availability

The original contributions presented in this study are included in the article/Supplementary Materials. Further inquiries can be directed to the corresponding authors.

## Supplementary Materials

The following supporting information can be downloaded at: https://www.mdpi.com/article/10.3390/children13050595/s1, Supplementary Table S1: Nutritional composition of the study samples.

## Abbreviations

The following abbreviations are used in this manuscript:

CM	Cow’s Milk
CMF	Cow’s Milk-based Infant Formula
DTT	Dithiothreitol
EFSA	European Food Safety Authority
GER	Gastroesophageal Reflux
GERD	Gastroesophageal Reflux Disease
GM	Goat Milk
GMF	Goat Milk-based Infant Formula
HGJ	Human Gastric Juice
HM	Human Milk
HMO	Human Milk Oligosaccharides
OPA	o-Phthaldialdehyde
PPI	Proton Pump Inhibitors
GDPR	General Data Protection Regulation
SDS-PAGE	Sodium Dodecyl Sulfate–Polyacrylamide Gel Electrophoresis
