# Supplementary material for: Impact of Gastric pH on Milk Protein Hydrolysis: A Pilot In Vitro Study Using Pediatric Human Gastric Juice in the Context of Infant Digestive Physiology
Source: Children (Basel). 2026 Apr 24;13(5):595. doi: 10.3390/children13050595 (PMC13204711; doi:10.3390/children13050595)
Supplement: Supplementary file 1 [file children-13-00595-s001.zip › children-4248103-supplementary.pdf]

children

MDPI

Supplementary Materials

Impact of Gastric pH on Milk Protein Hydrolysis: A Pilot In Vitro Study Using Pediatric Human Gastric Juice in the Context of Infant Digestive Physiology

Maria Del Nogal Avila [1], Marta Soria López [2], Isabel Sánchez-Vera [1], Rosa Plaza-Clavero [1], Daniel Cabello-Rivera [1], Karen Knipping [3] and Alejandro López-Escobar [4,*]

[1] Instituto de Medicina Molecular Aplicada (IMMA) Nemesio Díez, Facultad de Medicina, Universidad San Pablo-CEU, CEU Universities, Urbanización Montepríncipe, 28660 Boadilla del Monte, Spain; maria.nogalavila@ceu.es (M.D.N.A.); isanver@ceu.es (I.S.-V.); rosaplazaclavero@gmail.com (R.P.-C.); daniel.cabellorivera@ceu.es (D.C.-R.)
[2] Pediatrics Department, HM Hospitales, 28938 Madrid, Spain; soria.marta@gmail.com
[3] Ausnutria B.V., 8025 Zwolle, The Netherlands; karen.knipping@ausnutria.nl
[4] Facultad de Ciencias de la Salud, Universidad Internacional de la Rioja (UNIR), 28224 Pozuelo de Alarcón, Spain
* Correspondence: alejandro.lopezescobar@unir.net

Supplementary Table S1. Nutritional composition of the study samples (per 100 mL of reconstituted formula or mature human milk). Data for cow's milk-based infant formula (Blemil Plus Forte 1) and goat milk-based infant formula (Kabrita 1) are based on manufacturer specifications. Human milk values represent average ranges for mature milk (4 months postpartum) as reported in literature [14, 20]. α-La: α-lactalbumin; β-Lg: β-lactoglobulin.

Component	Cow's Milk Infant Formula	Goat Milk Infant Formula	Human Milk
**Energy (kcal)**	68	65	67
**Total Protein (g/100ml)**	1.18	1.3	1.0
**Whey:Casein Ratio**	60:40	60:40	60:40
**Major Caseins**	$\alpha_{s}1$, $\backslash\alpha_{s}2$, β	$\alpha_{s}1$ (low), $\backslash\alpha_{s}2$, β	β
**Major Whey Proteins**	β-Lg, α-La	β-Lg, α-La	α-La, Lactoferrin
**Total Fat (g/100ml)**	3.38	3.4	4.2
**Carbohydrates (g/100ml)**	7.62	7.2	6.5
**Protein Source**	Cow´s milk	Goat milk	Human donor
